# A Diet Pattern with More Dairy and Nuts, but Less Meat Is Related to Lower Risk of Developing Hypertension in Middle-Aged Adults: The Atherosclerosis Risk in Communities (ARIC) Study

**DOI:** 10.3390/nu5051719

**Published:** 2013-05-21

**Authors:** Lu-Chen Weng, Lyn M. Steffen, Moyses Szklo, Jennifer Nettleton, Lloyd Chambless, Aaron R. Folsom

**Affiliations:** 1 Division of Epidemiology and Community Health, University of Minnesota School of Public Health, 1300 South 2nd Street, Suite 300, Minneapolis, MN 55454, USA; E-Mails: wengx017@umn.edu (L.-C.W.); folso001@umn.edu (A.R.F.); 2 Department of Epidemiology, Johns Hopkins University, 615 N. Wolfe Street, Baltimore, MD 21205, USA; E-Mail: mszklo@jhsph.edu; 3 Department of Epidemiology and Disease Control, University of Texas Health Science Center-Houston, 1200 Herman Pressler, Houston, TX 77030, USA; E-Mail: Jennifer.A.Nettleton@uth.tmc.edu; 4 Department of Biostatistics, University of North Carolina-Chapel Hill, 3101 McGavran-Greenberg Hall, CB #7420, Chapel Hill, NC 27599, USA; E-Mail: wchambless@unc.edu

**Keywords:** diet pattern, healthy food score, hypertension, high normal blood pressure

## Abstract

Dietary intake among other lifestyle factors influence blood pressure. We examined the associations of an “*a priori*” diet score with incident high normal blood pressure (HNBP; systolic blood pressure (SBP) 120–139 mmHg, or diastolic blood pressure (DBP) 80–89 mmHg and no antihypertensive medications) and hypertension (SBP ≥ 140 mmHg, DBP ≥ 90 mmHg, or taking antihypertensive medication). We used proportional hazards regression to evaluate this score in quintiles (Q) and each food group making up the score relative to incident HNBP or hypertension over nine years in the Atherosclerosis Risk of Communities (ARIC) study of 9913 African-American and Caucasian adults aged 45–64 years and free of HNBP or hypertension at baseline. Incidence of HNBP varied from 42.5% in white women to 44.1% in black women; and incident hypertension from 26.1% in white women to 40.8% in black women. Adjusting for demographics and CVD risk factors, the “*a priori*” food score was inversely associated with incident hypertension; but not HNBP. Compared to Q1, the relative hazards of hypertension for the food score Q2–Q5 were 0.97 (0.87–1.09), 0.91 (0.81–1.02), 0.91 (0.80–1.03), and 0.86 (0.75–0.98); *p*_trend_ = 0.01. This inverse relation was largely attributable to greater intake of dairy products and nuts, and less meat. These findings support the 2010 Dietary Guidelines to consume more dairy products and nuts, but suggest a reduction in meat intake.

## 1. Introduction

Diet plays an important role in the modulation of blood pressure in hypertensive and normotensive adults [1,2,3,4,5,6,7,8,9,10,11,12]. Short-term randomized clinical trials, including the DASH (Dietary Approaches to Stop Hypertension) and OmniHeart, demonstrated that a combination diet rich in fish, lowfat dairy products, nuts and seeds, fruit, vegetables, legumes, and/or whole grains as well as lower in red and processed meat substantially lowered systolic and diastolic blood pressure in pre-hypertensive to moderately hypertensive white and black men and women [1,10,13]. In addition, a meta-analysis of 14 clinical trials conducted in the U.S., Europe, and Australia showed a significant blood pressure lowering effect of the Mediterranean Diet (MedDiet) pattern (a diet rich in olive oil, fish, legumes, nuts, vegetables, fruit, and moderate alcohol but low in meat and dairy products) [14]. These diet patterns have in common greater intakes of fruit, vegetables, legumes, nuts, and fish and low intake of meat; however, notable differences between these diet patterns are the inclusion of more lowfat dairy products in the DASH diet [1], whereas, a MedDiet includes more olive oil but fewer dairy products [14].

In observational studies, dietary pattern analysis has been useful in predicting diet-disease associations because a diet pattern captures total dietary intake instead of individual nutrient or food intakes [15]. However, study findings have been inconsistent possibly due to different study designs and study populations, method of diet assessment, or method of diet pattern derivation [11,16,17,18,19]. “*A posteriori*” derived dietary patterns, including the Western and Prudent patterns, are data driven and created through factor or cluster analysis [20]. “*A priori*” diet patterns or scores, such as the Healthy Eating Index (HEI) and Diet Diversity Score (DDS) [21,22], have been created based on dietary recommendations or other evidence. Food scores based on epidemiologic evidence, including a healthy food score has been inversely related to elevated blood pressure and incident myocardial infarction [19,23], while other diet and food scores have been unrelated to blood pressure [24]. Given the differential study results reported in dietary pattern analysis, there is also a need to elucidate the contribution of individual food groups in the diet pattern to lower or higher risk of developing high blood pressure.

We propose to examine the associations of an “*a priori*” healthy diet pattern [19] that is similar to a DASH-type diet pattern: a diet rich in dairy products, nuts, fruit, vegetables, and whole grain, but low in sugar-sweetened beverages and red/processed meat with the incidence of high normal blood pressure (HNBP) and hypertension in middle-aged African American and white men and women. We hypothesize that this healthy diet pattern will be inversely related to the development of HNBP and hypertension. In addition, we will determine whether certain food groups and usual number of servings of these foods explain a major proportion of the relation.

## 2. Materials and Methods

### 2.1. Population

The Atherosclerosis Risk in Communities (ARIC) study is a community-based prospective study investigating the etiology and natural history of atherosclerosis in middle age US adults [25]. Participants were recruited from four communities: Forsyth County, North Carolina; Jackson, Mississippi; suburbs of Minneapolis, Minnesota; and Washington County, Maryland. Among these communities, Caucasians were recruited from the Minneapolis suburbs (*n* = 3972), Washington County (*n* = 3975) and Forsyth County (*n* = 3531), but none in Jackson; while African Americans were recruited predominantly from Jackson (*n* = 3728) and some from Forsyth County (*n* = 483), Minneapolis (*n* = 22), and Washington County (*n* = 33). The total number of participants enrolled at the baseline exam (1987–1989) was 15,792 adults aged 45–64 years. Investigators reported less than 5% bias in prevalence estimates related to nonresponse for most of the measured characteristics [26]. Return rates of exams every three years were 92.7%, 85.6%, and 80.8% in 1990–1992 (Exam 2), 1993–1995 (Exam 3), and 1996–1998 (Exam 4), respectively. The ARIC protocol was approved by all participating Institutional review boards, and signed informed consents were obtained from all participants. For this analysis, participants were excluded who were not white or African American (*n* = 48), had hypertension at baseline (*n* = 5495), had missing data (*n* = 80), or had implausible energy intake (less than 500 and 700 kcals for women and men, respectively, and greater than 3500 and 4500 kcals for women and men, respectively) (*n* = 256), resulting in 9913 participants for this analysis.

### 2.2. Data Collection

Baseline information about demographic characteristics and lifestyle habits, including smoking and physical activity, were collected by trained interviewers. Height was measured without shoes to the nearest 1 cm and weight measured in light clothing to the nearest 0.1 kg on a beam balance scale. Body mass index (BMI) was calculated by weight in kilograms divided by height in meters squared. Smoking status was categorized as current smoker, former smoker, and never smoker. A modified Baecke physical activity questionnaire assessed leisure time physical activity [27]. A sport index was created representing leisure time physical activity and scored from 1 to 5. Fasting blood samples were obtained at baseline to measure lipid and glucose concentrations. Participants were classified as having diabetes if they had a fasting glucose ≥126 mg/dL (7.0 mmol/L), a non-fasting glucose ≥200 mg/dL (11.1 mmol/L), reported that a physician had told them they had diabetes or reported taking medication for diabetes within two weeks preceding their examination.

### 2.3. Dietary Assessment

The ARIC study used a modified 66-item interviewer-administered food-frequency questionnaire (FFQ) to assess dietary intake at baseline and Exam 3 [28]. Modifications included additional fish categories (dark, oily fish; lean, white fish) and vitamin supplement information (omega-3 fatty acid supplements), and information about type of cooking fat used (*i.e.*, butter, margarine, vegetable oil, vegetable shortening, lard, bacon grease). Study participants were also asked to report other foods not queried and these were coded. Nine frequency categories from almost never to over six times a day were asked by the FFQ, and standard portion sizes and food models were provided to evaluate the amount of consumption. Use of salt added in cooking and at the table as well as brand name of usual cold breakfast cereal consumption were also obtained. 

The Healthy Food Score (HFscore), adapted from Steffen *et al.* [19] was based on published evidence of diet-disease relations [1,4,5,11,12,19] and created by summing the scores of food groups. Specifically, the following food groups were created: dairy (combined low-fat and high fat milk, cheese, yogurt, ice cream), vegetables, fruit (without juice), fruit juice, legumes, refined grain, whole grain, nuts, fish, meat (combined poultry, processed meat, beef, pork, and lamb), diet beverages, sugar-sweetened beverages, and coffee and tea. Daily food intake was calculated using frequency and standard portion size for each food group. Daily servings of food groups were then categorized into quintiles, except alcohol intake, legume, and beverages. Each quintile of food group intake was assigned a score: 0–4. For dairy, vegetables, fruit (without juice), fruit juice, refined grain, whole grain, nuts, and fish, scores were assigned in order (Quintile 1 = 0, Quintile 2 = 1, Quintile 3 = 2, Quintile 4 = 3, Quintile 5 = 4); for meat, the score was the reverse. Due to the limited range of intake, scoring for intake of legumes was 0, 1, and 2, if daily intake was 0, <1, and ≥1 serving, respectively. The score was reversed for diet beverages and sugar-sweetened beverages: 2, 1, and 0 for 0, >0 to <1, and one or more servings usually consumed per day, respectively [29]. Daily coffee and tea intake was scored in five categories from 0 to 4, for 0, >0 to ≤2, >2 to ≤4, >4 to ≤6, and >6 cups per day, respectively [30]. For alcohol intake, a score of 4 was assigned to the men who consumed between 10 and 50 g per day and to women who consumed between 5 and 30 g per day; otherwise a score of 0 was assigned [31]. 

### 2.4. Blood Pressure Measurement

The average of the second and third sitting blood pressure readings as measured by random-zero sphygmomanometer (RZ) was used in Exams 1–3, while the mean blood pressure as measured by the RZ in Exam 4 included the only two blood pressure measurements obtained. High normal blood pressure was defined as systolic blood pressure greater than or equal to 120 mmHg and less than 140 mmHg and/or diastolic blood pressure greater than 80 mmHg and less than 90 mmHg and no use of antihypertensive medication. Hypertension was defined as greater than or equal to 140 mmHg systolic, 90 mmHg diastolic, or use of antihypertensive medication. Follow-up time was terminated for participants who developed incident high-normal blood pressure (yes/no) or incident hypertension (yes/no). Because actual time to diagnosis is unknown, the time to blood pressure events was interval censored by the follow-up visit schedule [32]. Follow-up time for those with neither endpoint ended at the last attended exam or at the end of study (Exam 4).

High normal blood pressure and hypertension were defined as independent endpoints. Participants with normal blood pressure at baseline who developed high normal blood pressure, but not hypertension, were included in the analysis of the high normal blood pressure outcome (*n* = 5260). However, participants with normal or high normal blood pressure at baseline who developed hypertension were included in the hypertension outcome only (*n* = 9913).

### 2.5. Statistical Methods

All analyses were conducted using SAS (version 9.2, SAS Institute Inc, Cary, NC, USA). Baseline characteristics are described as mean ± SD or frequencies. Cumulative average diet, which has been shown to increase the precision of dietary data, was used to create food groups and the HFscore [33]. Specifically, the time-varying dietary covariates were modeled as follows: between baseline and Exam 3, dietary exposures were based on diet as measured at the baseline exam; after Exam 3, dietary exposures were based on the mean of baseline and Exam 3 dietary intakes. Cox proportional hazards regression analysis evaluated the relation of incident high-normal blood pressure or hypertension across quintiles of the HFscore. Three models were developed including factors that may confound the relation between diet and blood pressure: Model 1 adjusted for baseline age, sex, race, field center, education, and energy intake; Model 2 adjusted for Model 1 plus baseline physical activity and smoking status; and Model 3 further adjusted for baseline diabetes mellitus, BMI and BMI change (BMI is a potential mediator of diet and blood pressure). We also examined the relation between each food group relative to hypertension. To further determine if the relation between the HFscore and hypertension may be attributed to one or more food groups, we removed selected food groups from the HFscore to create a partial HFscore. The relations between the food group of interest and hypertension were evaluated adjusting for Model 2 confounders plus the partial HFscore. Effect modification by sex and race was tested.

## 3. Results

In this ARIC sample free of baseline high normal blood pressure (*n* = 5260) and hypertension (*n* = 9913), the mean ± SD age at baseline was 54.0 ± 5.7 years in men and 53.0 ± 5.6 years in women. The mean ± SD systolic and diastolic blood pressures at baseline for the total population were 114 ± 12 mmHg and 70 ± 9 mmHg, respectively (Table 1); by Exam 4 the mean systolic blood pressure increased to 123 ± 17 mmHg, while the mean diastolic blood pressure remained 70 ± 10 mmHg. Among the four sex-ethnic groups, white women had the lowest mean systolic and diastolic blood pressures at baseline. Over nine years of follow-up, 1663 participants developed high normal blood pressure and 2853 participants developed hypertension. The incident proportions of high normal blood pressure did not vary much across gender-ethnic groups. However, blacks, particularly women, had the highest incidence of hypertension compared with whites.

**Table 1:** Baseline and Exam 4 blood pressure levels and incidence of high normal blood pressure or hypertension among ARIC participants, 1987–1998.

ARIC participants	All (*n* = 9913)	Black men (*n* = 700)	White men (*n* = 3759)	Black women (*n* = 1092)	White women (*n* = 4362)
Systolic/diastolic BP at baseline, mmHg *	*n* = 9913 114 ± 12/70 ± 9	*n* = 700 116 ± 0.7/73 ± 0.5	*n* = 3759 115 ± 0.2/72 ± 0.2	*n* = 1092 116 ± 0.7/70 ± 0.5	*n* = 4362 112 ± 0.2/68 ± 0.2
Systolic/diastolic BPat Exam 4, mmHg *	*n* = 7736 123 ± 17/70 ± 10	*n* = 461 125 ± 1.3/71 ± 0.7	*n* = 2974 122 ± 0.4/71 ± 0.2	*n* = 751 127 ± 1.2/70 ± 0.7	*n* = 3550 122 ± 0.4/68 ± 0.2
Incident high-normal BP, *n* (%) ^†^	2642(42.9)	132(43.0)	988(47.7)	232(44.1)	1290(42.5)
Incident hypertension, *n* (%) ^‡^	2853(28.8)	242(34.6)	1027(27.3)	445(40.8)	1139(26.1)

* All values are mean (SE) adjusted for age, center, education; except for all (total population, which is the mean ± SD); ^†^ Incident high normal blood pressure (*n* = 6153) is defined as a systolic blood pressure (BP) ≥120 but <140 mmHg or a diastolic BP ≥80 but <90 mm Hg and not using anti-hypertension medication; ^‡^ Incident hypertension (*n* = 9913) is defined as systolic BP ≥140 mmHg, diastolic BP ≥90 mmHg, or the use of antihypertensive medication.

Participants who were categorized with a lower HFscore (Quintile 1) were more likely to be younger, African American, current smokers, physically inactive, and have less education than those in Quintile 5 (Table 2). Intakes of fiber, calcium, vitamin D, alcohol, and servings of dairy, fish and seafood, fruit and vegetables, fruit juice, legumes, whole grain, and refined grain were higher, while % kcal from fat and servings of meat were lower across successive quintiles of HFscore intake.

**Table 2:** Unadjusted mean (SD) and frequency (%) characteristics at baseline across Healthy Food Score quintiles among African American and white adults who were not hypertensive at baseline, ARIC (*n* = 9913), 1987–1989.

	Healthy Food Score (HFscore) Quintiles									
Characteristics	Q1 (*n* = 2127)		Q2 (*n* = 1568)		Q3 (*n* = 2582)		Q4 (*n* = 1662)		Q5 (*n*=1974)	
*Mean HFscore*										
Age, year (SD)	52.6	(5.5)	53.4	(5.6)	53.4	(5.7)	53.8	(5.7)	54.1	(5.7)
Blacks, *n* (%)	567	(26.7)	317	(20.2)	447	(17.3)	256	(15.4)	205	(10.4)
Females, *n* (%)	1197	(56.3)	833	(53.1)	1380	(53.4)	939	(56.5)	1105	(56.0)
<High school, *n* (%)	619	(29.1)	350	(22.3)	459	(17.8)	231	(13.9)	233	(11.8)
Current smoking, *n* (%)	699	(32.9)	430	(27.5)	672	(26.0)	409	(24.6)	475	(24.1)
Physical activity score (SD)	2.3	(0.7)	2.4	(0.8)	2.5	(0.8)	2.6	(0.8)	2.7	(0.8)
Body Mass Index, kg/m^2^ (SD)	27.2	(5.0)	26.8	(4.8)	26.8	(4.8)	26.6	(4.7)	26.1	(4.4)
Systolic blood pressure, mmHg (SD)	114.2	(12.3)	113.6	(12.6)	113.7	(12.5)	113.6	(12.1)	113.0	(12.2)
Diastolic blood pressure, mmHg (SD)	70.7	(8.8)	70.0	(8.6)	69.9	(8.7)	69.8	(8.5)	69.3	(8.5)
*Nutrient intake, unit per day (SD)*										
Energy (kcal)	1322	(528)	1478	(523)	1632	(575)	1773	(571)	2013	(596)
% kcal from fat	35.1	(7.5)	34.1	(6.5)	33.0	(6.4)	32.2	(6.3)	31.5	(5.9)
Dietary fiber (g)	10.9	(5.0)	14.3	(5.2)	17.2	(6.8)	20.1	(7.3)	24.5	(8.8)
Calcium (mg)	418.3	(234.0)	558.7	(306.9)	671.8	(344.8)	785.0	(366.5)	929.2	(397.2)
Vitamin D (IU)	131.4	(89.5)	183.6	(126.4)	220.5	(129.6)	258.3	(132.9)	311.5	(155.0)
Alcohol (g)	4.8	(15.0)	4.7	(11.6)	5.6	(12.1)	6.5	(13.0)	8.3	(12.1)
*Food Intake, servings/day (SD)*										
Dairy	0.96	(0.85)	1.38	(1.15)	1.72	(1.31)	2.04	(1.35)	2.43	(1.47)
Meat	1.42	(0.81)	1.40	(0.79)	1.38	(0.83)	1.37	(0.79)	1.38	(0.81)
Fish and seafood	0.18	(0.19)	0.25	(0.29)	0.30	(0.28)	0.36	(0.30)	0.49	(0.36)
Nuts	0.05	(0.12)	0.08	(0.17)	0.12	(0.29)	0.16	(0.27)	0.26	(0.36)
Fruit and vegetables	2.04	(1.24)	2.80	(1.37)	3.50	(1.80)	4.15	(1.97)	5.15	(2.26)
Fruit juice	0.24	(0.43)	0.39	(0.51)	0.51	(0.58)	0.64	(0.61)	0.81	(0.71)
Legumes	0.20	(0.21)	0.24	(0.24)	0.27	(0.31)	0.30	(0.28)	0.37	(0.35)
Whole grain products	0.61	(0.75)	0.98	(1.03)	1.27	(1.15)	1.67	(1.35)	1.98	(1.30)
Refined grain products	2.10	(1.67)	2.30	(1.70)	2.43	(1.82)	2.46	(1.69)	2.65	(1.67)
Sugar-sweetened beverage	0.79	(1.20)	0.57	(0.91)	0.50	(0.82)	0.43	(0.77)	0.37	(0.72)
Diet soda	0.56	(1.07)	0.56	(1.00)	0.51	(0.96)	0.52	(0.93)	0.46	(0.87)

There was no relation for the risk of developing high normal blood pressure across quintiles of HFscore (*p*_trend_ = 0.63) (Table 3, Model 2), while the HFscore was inversely associated with development of hypertension (Table 4, Full HFscore models). Participants categorized in the highest HFscore quintile were at 14% lower risk of incident hypertension (Model 2; *p*_trend_ = 0.01). After adjusting this model for BMI and diabetes at baseline and change in BMI, the relation was slightly attenuated (Model 3; *p*_trend_ = 0.06).

**Table 3:** Hazard ratios (HR) and 95% confidence intervals (95% CI) for incident high normal blood pressure* across Healthy Food Score quintiles, ARIC (*n* = 5260), 1987–1998.

	Healthy food score (HFscore) quintiles					
	Q1 (*n* = 1107)	Q2 (*n* = 860)	Q3 (*n* = 1333)	Q4 (*n* = 879)	Q5 (*n* = 1081)	*p* for trend ^†^
Mean HFscore	15.4	20.1	23.5	27.0	31.7	
Model 1 HR (95% CI)	1.0	1.04 (0.89–1.23)	1.11 (0.95–1.31)	1.03 (0.86–1.23)	1.06 (0.89–1.25)	0.63
Model 2 HR (95% CI)	1.0	1.04 (0.88–1.22)	1.11 (0.94–1.31)	1.04 (0.87–1.24)	1.06 (0.89–1.26)	0.63
Model 3 HR (95% CI)	1.0	1.04 (0.88–1.22)	1.11 (0.94–1.31)	1.03 (0.87–1.23)	1.08 (0.91–1.29)	0.90

* High normal blood pressure is defined as systolic blood pressure ≥120 mmHg but <140 mmHg, diastolic blood pressure ≥80 mmHg but <90 mmHg; ^†^ Linear trend across quintiles was tested by assigning the median intake of each quartile to each individual in that quartile and treating it as a continuous variable; Model 1 adjusted for baseline age, sex, race, education, center, energy intake, and added salt (participant response (frequency) to the salt question “how often is salt or salt-containing seasoning such as garlic salt, onion salt, soy sauce, or Accent added to your food in cooking?”; Model 2 adjusted for Model 1 and baseline physical activity and smoking; Model 3 adjusted for Model 2 and baseline BMI, BMI change, and diabetes mellitus.

**Table 4:** Hazard ratios (HR) and 95% confidence intervals (95% CI) for incident hypertension* across Healthy Food Score quintiles, ARIC (*n* = 9913), 1987–1998.

	Healthy food score (HFscore) quintiles					
	Q1 (*n* = 2127)	Q2 (*n* = 1568)	Q3 (*n* = 2582)	Q4 (*n* = 1662)	Q5 (*n* = 1974)	*p* for trend ^†^
Mean HFscore	15.4	20.0	23.5	27.0	31.7	
*Full* *Health food* *score models*						
Model 1 HR (95% CI)	1.0	0.97 (0.87–1.09)	0.91 (0.81–1.02)	0.90 (0.79–1.02)	0.85 (0.74–0.96)	0.006
Model 2 HR (95% CI)	1.0	0.97 (0.87–1.09)	0.91 (0.81–1.02)	0.91 (0.80–1.03)	0.86 (0.75–0.98)	0.01
Model 3 HR (95% CI)	1.0	0.98 (0.88–1.10)	0.92 (0.82–1.04)	0.92 (0.81–1.05)	0.90 (0.79–1.02)	0.06
*Partial Healthy food score models (Model 2)*						
(a) HFscore without meat						
Partial HFscore	1	1.00 (0.89–1.13)	0.96 (0.85–1.08)	0.94 (0.83–1.07)	0.95 (0.83–1.09)	0.33
Meat **	1	1.01 (0.9–1.14)	0.90 (0.79–1.02)	0.88 (0.77–1.01)	0.82 (0.71–0.94)	0.002
(b) HFscore without dairy						
Partial HFscore	1.0	0.99 (0.88–1.11)	0.94 (0.82–1.07)	0.92 (0.82–1.04)	0.92 (0.81–1.06)	0.14
Dairy	1.0	0.96 (0.85–1.07)	0.95 (0.84–1.07)	0.92 (0.81–1.04)	0.85 (0.75–0.97)	0.01
(c) HFscore without nuts						
Partial HFscore	1.0	0.95 (0.85–1.07)	0.88 (0.78–1.00)	0.94 (0.82–1.07)	0.89 (0.78–1.02)	0.10
Nuts	1.0	1.11 (0.98–1.27)	0.96 (0.87–1.07)	0.92 (0.81–1.04)	0.87 (0.77–0.97)	0.002
(d) HFscore without dairy, meat, & nuts						
Partial HFscore	1.0	0.98 (0.87–1.10)	0.93 (0.82–1.04)	0.97 (0.86–1.10)	1.03 (0.90–1.18)	0.90
Meat	1.0	1.03 (0.91–1.16)	0.92 (0.81–1.04)	0.90 (0.78–1.02)	0.84 (0.73–0.97)	0.009
Dairy	1.0	0.96 (0.85–1.07)	0.95 (0.84–1.07)	0.92 (0.82–1.04)	0.86 (0.75–0.98)	0.02
Nuts	1.0	1.12 (0.98–1.27)	0.96 (0.87–1.07)	0.91 (0.81–1.03)	0.86 (0.77–0.97)	0.002

* Hypertension was defined as a systolic blood pressure ≥140 mmHg, a diastolic blood pressure ≥90 mmHg, or taking antihypertensive medication; ** The scoring for meat was the reverse of dairy, for example, less meat consumed = Q5, while more dairy = Q5; ^†^ Linear trend across quintiles was tested by assigning the median intake of each quartile to each individual in that quartile and treating it as a continuous variable; Model 1 adjusted for baseline age, sex, race, education, center, energy intake, and added salt (participant response (frequency) to the salt question “how often is salt or salt-containing seasoning such as garlic salt, onion salt, soy sauce, or Accent added to your food in cooking?”; Model 2 adjusted for Model 1 and baseline physical activity and smoking; Model 3 adjusted for Model 2 and baseline BMI, BMI change, and diabetes mellitus.

To determine which food groups in the HFscore that contributed most to the risk of developing hypertension, we examined individual food groups relative to incident hypertension (Supplemental Table 1). Greater intake of dairy products and nuts and lower intake of meat were significantly and inversely related to hypertension; however, no other food group was related. Three partial HFscore models were created by removing the “dairy”, “nuts” and/or “meat” groups from the HFscore variable to examine the independent relations of dairy, nuts, meat, and the partial HFscores on the risk of developing hypertension (Table 4; Partial HFscore models). We found that the partial HFscores did not remain significant when each of “meat”, “dairy”, or “nuts” was omitted from the HFscore (Partial HFscore models a, b, and c), but these partial HFscores were still inversely related to hypertension incidence. When the three food groups “meat’, “dairy”, and “nuts” were all omitted from the HFscore (Partial HFscore Model d), the combination of the remaining food groups was not related to hypertension (*p*_trend_ = 0.90); however, compared to Quintile 1 intake, consuming one or less daily serving of meat, more than two daily servings of dairy products, and about one quarter serving of nuts per day (Quintile 5 intake) was independently related to lower risk of developing hypertension. Adjusting the model for calcium and vitamin D enhanced the inverse association for dairy (data not shown). Race or gender did not modify the relations between the HFscore and incident high normal blood pressure or hypertension.

## 4. Discussion

The HFscore was inversely related to the risk of developing hypertension, but not HNBP, in middle-aged African American and white men and women. Moreover, in studying the components of the diet pattern, the inverse relation with hypertension was largely attributable to greater intakes of dairy products and nuts, and lower intake of meat. Specifically, study participants with the lowest risk of developing hypertension consumed more than two daily servings of dairy products, at least one serving of nuts per week, and one or fewer daily servings of meat, in addition to the combination of greater intakes of fruit, vegetables, whole grains, legumes, and fish and lower intake of sugar sweetened beverages.

Our findings were consistent with those observed in the CARDIA study of young African American and white men and women, the study from which the HFscore used in ARIC was adapted [19]. Even though different dietary assessment instruments were used—the 66-item FFQ [28] in ARIC and the Diet History questionnaire [34] in CARDIA—similar food groups and the same scoring scheme were used to create the food score in each study. One diet quality score, the Recommended Foods Score, which includes only foods recommended for health but not other foods consumed in the rest of the diet, has been related to lower blood pressure [24]. Other diet quality scores, such as the Dietary Diversity Score or the Healthy Eating Index (HEI), are also based on dietary food recommendations, but have had limited success in explaining disease risk [24,35]. The MedDiet pattern, based on the traditional diet of the Mediterranean area and which features olive oil consumption, has consistently been inversely related to a variety of chronic diseases [36], including high blood pressure [37], despite the use of a variety of study designs, study populations, and diet assessment instruments. Although the DASH diet successfully reduced blood pressure in short-term clinical trials [1], not all observational studies have shown a long-term beneficial relation between a DASH-like diet and development of hypertension in longitudinal studies [38]. Finally, numerous “data driven” dietary patterns derived by factor or cluster analysis have been inconsistently related to change in blood pressure or incident hypertension. The inconsistent findings may potentially be explained by differences in the method of diet assessment, the study population and the geographic location of each study (e.g., food availability) [18,39,40,41].

Our findings demonstrate a lower risk of hypertension with higher intake of dairy products and nuts, but lower intake of meat, after adjusting for the rest of the diet pattern and other confounding factors. Inconsistent results have been reported for studies about dairy products, whether total dairy, high-fat or lowfat dairy, relative to elevated blood pressure (defined as SBP ≥130 mmHg and/or DBP ≥85 mmHg) [19] and high blood pressure [11,42,43,44]. Dietary patterns with higher intake of dairy products, may lower blood pressure by modulating insulin secretion [45], although the evidence is inconsistent [46]. Another potential blood pressure lowering mechanism may be the proteins in milk that include both casein and whey which contain blood pressure lowering properties [47]. In addition, dairy products contain vitamin D, calcium magnesium, and individual fatty acids 15:0 and 17:0 which correlate inversely with cardiovascular risk factors [48,49]. Alternatively, dietary calcium may suppress 1,25-dihydroxyvitamin D concentrations, thereby decreasing vascular smooth muscle cell intracellular calcium and also reducing peripheral vascular resistance and blood pressure [50]. Thus, we speculate that a beneficial effect of dairy products on blood pressure may be due to the myriad of nutrients and dietary compounds in dairy products.

Generally, greater meat consumption has been associated with higher risk of developing elevated or high blood pressure among a variety of populations [11,19,51,52]. In contrast to dairy products, meat contains more saturated fatty acid and cholesterol, which may increase risk of coronary heart disease [53]. In a meta-analysis of total protein intake relative to blood pressure, eight cross-sectional studies showed an inverse relation, while four prospective studies reported no relation between total protein intake and blood pressure [54]. Further, both plant protein and protein from meat sources have been inconsistently related to blood pressure [54,55]. In addition, red meat contains high levels of heme iron, which has an unfavorable effect on blood pressure levels [56]. Moreover, processed meat contains considerable salt/sodium, a known risk factor for hypertension. Otherwise, higher consumption of meat may be a factor contributing to weight gain, which is positively associated with elevated blood pressure [57]. Our results showed that adjustment for BMI attenuated the HFscore- hypertension relation, which further supports the mediating effect of BMI on blood pressure.

Finally, our study adds to the accumulating evidence that nut consumption has a blood pressure lowering effect [58]. Nuts are comprised of several components that have been related to lower blood pressure, including plant protein, fiber, antioxidants, monounsaturated and polyunsaturated fatty acids, polyphenols, and the minerals magnesium and calcium.

There are a few limitations to our study. Because blood pressure fluctuates during the day and over time, we captured blood pressure at only one sitting during each exam; however, participants had the opportunity for four blood pressure measurements between 1987–1989 and 1996–1998. Second, even though the FFQ used to assess dietary intake included only 66-items, which may misclassify food intake somewhat, study participants had the opportunity to report other foods not queried, which were coded and included in analysis. Nevertheless, daily nutrient intake may be underestimated; however, food intake is most likely correctly ranked. While acknowledging these limitations, the FFQ has been previously tested for validity and reliability of diet patterns yielding correlations ranging from 0.45 to 0.74 between food records and two FFQs [28]. Finally, statistical models were adjusted for numerous potential factors which may confound the relation between dietary intake and blood pressure category; however, residual confounding may still exist.

The strengths of this study include its prospective study design, high participation rate, and the standardized procedure for measuring blood pressure at four clinic exams. Further, dietary intake was measured on two occasions [33]. In addition, few, if any, cohort studies have previously examined the relation between the HFscore and high normal blood pressure in Caucasian and African American populations.

## 5. Conclusions

These findings are consistent with the 2010 U.S. Dietary Guidelines for Americans [59], and suggest that a healthy diet pattern is related to a lower risk of developing hypertension, especially a diet rich in dairy and nut products, but low in meat. 

## Supplementary Files

Supplementary File 1 Supplementary Information (PDF, 43 KB)
