# Supplementary material for: A Diet Pattern with More Dairy and Nuts, but Less Meat Is Related to Lower Risk of Developing Hypertension in Middle-Aged Adults: The Atherosclerosis Risk in Communities (ARIC) Study
Source: Nutrients. 2013 May 21;5(5):1719–33. doi: 10.3390/nu5051719 (PMC3708346; doi:10.3390/nu5051719)
Supplement: Supplementary File 1 — Supplementary Information (PDF, 43 KB) [file nutrients-05-01719-s001.pdf]

Supplementary Information

Table S1. Hazard ratios (95% confidence interval) of the relation between food group intake and risk of developing hypertension, ARIC, n = 9913.

	Quintiles of food group intake (servings/day)					
	**Q1 (low)**	**Q2**	**Q3**	**Q4**	**Q5 (high)**	$p_{\text{for trend}}$
HFScore	1	0.96 (0.86–1.08)	0.89 (0.79–1.02)	0.92 (0.82–1.03)	0.84 (0.73–0.96)	0.01
Food group						
Vegetables	1	0.97 0.86–1.09)	0.94 (0.83–1.06)	0.97 (0.85–1.09)	0.96 (0.84–1.09)	0.59
Fruit	1	1.06 (0.94–1.19)	0.98 (0.87–1.10)	1.08 (0.96–1.22)	1.06 (0.93–1.20)	0.37
Legumes [1]	1	1.01 (0.85–1.21)	1.00 (0.73–1.35)			0.94
Whole grain	1	1.04 (0.93–1.17)	1.03 (0.91–1.16)	1.00 (0.89–1.13)	0.99 (0.87–1.12)	0.61
Refined grain	1	1.04 (0.92–1.16)	0.99 (0.88–1.12)	1.03 (0.91–1.17)	1.00 (0.87–1.15)	0.92
Nuts	1	1.11 (0.98–1.26)	0.96 (0.87–1.07)	0.91 (0.81–1.03)	0.86 (0.77–0.97)	0.001
Dairy	1	0.95 (0.85–1.07)	0.94 (0.84–1.06)	0.91 (0.81–1.03)	0.85 (0.74–0.96)	0.009
Fish	1	1.01 (0.90–1.14)	1.04 (0.92–1.18)	1.06 (0.94–1.19)	1.09 (0.97–1.23)	0.11
Meat [2]	1	1.01 (0.90–1.13)	0.90 (0.79–1.02)	0.87 (0.76–1.00)	0.82 (0.71–0.94)	0.002
Coffee, tea [3]	1	1.02 (0.88–1.17)	1.01 (0.87–1.17)	0.91 (0.77–1.08)	0.83 (0.68–1.02)	0.01
Alcohol [4]	1	1.07 (0.98–1.18)				0.72
Sugar sweetened beverages [5]	1	0.90 (0.81–0.99)	0.98 (0.86–1.11)			0.17
Diet beverages [5]	1	0.89 (0.80–0.98)	0.83 (0.75–0.92)			<0.001

[1] Legumes are categorized as Q1 = 0, 0 ≤ Q2 < 1, Q3 ≥ 1 servings/day; [2] Meat intake is reverse scoring: Q1 = high and Q5 = low intake; [3] Coffee, tea categories: Q1 = 0; 0 ≤ Q2 < 2; 2 ≤ Q3 < 4; 4 ≤ Q4 < 6; Q5 ≥ 6 cups per day; [4] Alcohol categories defined by gender: Q2—for male: 10–50 g; for female: 5–30 g; Q1—other; [5] Sugar sweetened beverages and diet beverage categories defined as Q1 ≥ 1, Q2 < 1, Q3 = 0.
